# Susceptibility of human *Plasmodium knowlesi* infections to anti-malarials

**DOI:** 10.1186/1475-2875-12-425

**Published:** 2013-11-19

**Authors:** Farrah A Fatih, Henry M Staines, Angela Siner, Mohammed Atique Ahmed, Lu Chan Woon, Erica M Pasini, Clemens HM Kocken, Balbir Singh, Janet Cox-Singh, Sanjeev Krishna

**Affiliations:** 1 Division of Clinical Sciences, Centre for Infection and Immunity, St. George’s, University of London, London SW17 0RE, UK; 2 Malaria Research Centre, University Malaysia Sarawak, Kuching 93150, Malaysia; 3 Pathology Laboratory, Hospital Sarikei, Sarikei 96100, Malaysia; 4 Biomedical Primate Research Centre, Lange Kleiweg 161, GJ Rijswijk, The Netherlands; 5 School of Medicine, University of St Andrews, Medical and Biological Sciences Building, North Haugh, St Andrews KY16 9TF, UK

**Keywords:** Artemisinin, Artemether, Artesunate, Dihydroartemisinin, DHA, Chloroquine, Mefloquine, Malaria

## Abstract

**Background:**

Evidence suggests that *Plasmodium knowlesi* malaria in Sarawak, Malaysian Borneo remains zoonotic, meaning anti-malarial drug resistance is unlikely to have developed in the absence of drug selection pressure. Therefore, adequate response to available anti-malarial treatments is assumed.

**Methods:**

Here the *ex vivo* sensitivity of human *P. knowlesi* isolates in Malaysian Borneo were studied, using a WHO schizont maturation assay modified to accommodate the quotidian life cycle of this parasite. The *in vitro* sensitivities of *P. knowlesi* H strain adapted from a primate infection to *in vitro* culture (by measuring the production of *Plasmodium* lactate dehydrogenase) were also examined together with some assays using *Plasmodium falciparum* and *Plasmodium vivax*.

**Results:**

*Plasmodium knowlesi* is uniformly highly sensitive to artemisinins, variably and moderately sensitive to chloroquine, and less sensitive to mefloquine.

**Conclusions:**

Taken together with reports of clinical failures when *P. knowlesi* is treated with mefloquine, the data suggest that caution is required if using mefloquine in prevention or treatment of *P. knowlesi* infections, until further studies are undertaken.

## Background

From its natural simian hosts in Southeast Asia, *Plasmodium knowlesi* has emerged as a significant human pathogen, particularly in Malaysian Borneo [1-3]. Human *P. knowlesi* infections cause febrile illnesses that can rapidly progress to severe and sometimes fatal outcomes [4]. Ominously, the incidence of *P. knowlesi* malaria is increasing in geographic areas where *Plasmodium falciparum* and *Plasmodium vivax* are coming under control, thereby threatening the aim of eliminating malaria [5]. Determining the efficacy of conventional anti-malarials against *P. knowlesi* is a priority, particularly as there are no reports of anti-malarials assessed against human isolates of *P. knowlesi ex vivo*.

Here, the drug sensitivity profiles of *P. knowlesi* isolates obtained from patients being recruited into a study of the pathophysiology of knowlesi malaria in an endemic area of Sarawak, Malaysian Borneo were investigated. Currently, the WHO recommends artemisinin-based combination therapy (ACT) as first-line treatment for malaria in most endemic areas, so artemisinin and its clinically useful derivatives artesunate, dihydroartemisinin (DHA) and artemether were tested. Mefloquine, used as a partner drug in certain artemisinin-based combinations and in prophylaxis against malaria, and chloroquine that is recommended for treatment of *Plasmodium malariae* (the species which *P. knowlesi* is often confused with when diagnosed by microscopy) were also included. In addition, results from *P. falciparum* and *P. vivax* studied contemporaneously are presented, together with those obtained with the H strain laboratory isolate of *P. knowlesi* (cultured *in vitro* in rhesus erythrocytes) to confirm the methodologies used. Insights into the drug susceptibility patterns of this important emerging parasite, may prove useful in guiding the best choice of anti-malarial treatment regimens for *P. knowlesi* infection.

## Methods

### Patient recruitment

*Plasmodium* isolates were obtained from patients presenting to hospitals in Sarikei and Sibu. Informed written consent was obtained from all patients entered into this study, which was approved by the Malaysian Ministry of Health’s Medical Research and Ethics Committee, and the Sarawak State Planning Unit. Infecting species was confirmed by *Plasmodium* species-specific nested-PCR assays [6] and only patients with single species infections were retained in the study.

### Blood collection and *ex vivo* parasite development in growth assays

Pre-treatment venous blood from each patient was collected into EDTA. Parasitaemia and the asexual stage of development were determined by Giemsa-stained thin film microscopy. Whole blood (~2.5 ml) was washed twice without centrifugation to avoid haemolysis, before resuspending in RPMI 1640 complete medium supplemented with 20 mM D-glucose, 40 mM HEPES, 25 mg/l gentamicin sulphate, and 15% *v/v* human AB plasma with 0.2 mM hypoxanthine.

Growth inhibition by anti-malarials was assessed by quantifying schizont maturation using an adapted WHO Mark III assay [7]. Species of *Plasmodium* tested and the time lag in maturation seen previously *ex vivo* were allowed for in these assays [8]. While more complex counting procedures have been used to study *P. vivax* parasites that, like *P. knowlesi*, have mature parasites present in circulating blood [9], only the timing of assays was altered. This is because the *P. knowlesi* isolates contained predominantly immature parasites (Table 1) and results from a parallel study on tightly synchronised immature laboratory *P. knowlesi* H strain parasites with artesunate using the *p*LDH (see below) and Mark III assays were comparable with each other and with the data derived from isolates (see Results).

**Table 1:** Patient isolate data

**Isolate**	**Species**	**% starting parasitaemia**	**% schizont at start of assay**	**Duration of *in vitro *** **development (h)**	**% schizont at end of assay**
P0002	*Pk*	0.4	15	12	80
P0003	*Pk*	0.6	10	12	86
P0006	*Pk*	0.8	33	17	53
P0009	*Pk*	7.0^a^	17	15	94
P0010	*Pk*	0.8	6	12	63
P0011	*Pk*	1.3	8	14	54
P0007	*Pf*	2.0^a^	0	31	50
P0001	*Pv*	0.5	20	18	58
P0013	*Pv*	0.5	30	29	60

^a^Above Mark III assay cut-off (for examination of *P. falciparum* mono-infections), assuming a parasitaemia of 1.6% equates to approximately 80,000 parasites per μl of blood, and was used undiluted. Note that in both cases good schizont development was observed.

For each isolate, the species, parasitaemia and change in development over time are presented.

Aliquots (100 μl) of no drug control and serial dilutions of the anti-malarial compounds in culture medium (final concentrations of 0.25 to 25 nM for artemisinin and derivatives and 1.25 nM to 1 μM for chloroquine and mefloquine) were dispensed into 96-well plates, and parasites were added (100 μl at 2% haematocrit). Incubation was in 5% O_2_, 5% CO_2_, 90% N_2_ at 37°C until the majority of parasites reached the schizont stage (with at least 3 nuclei after 12–17 h for *P. knowlesi*). Monitoring of maturation was undertaken every 2 h by examination of fixed, Giemsa stained thin blood films, taken from a parallel culture to those of the drug exposed cultures. When at least half of the parasites in the monitoring culture had reached the schizont stage of development, the drug exposed cultures and their controls were harvested, as thick films on glass slides, and examined. The duration of *in vitro* development for each isolate (as determined above), which also equates to the time of drug exposure, is given in Table 1. Thick films were fixed by air drying for at least 24 h, stained with Giemsa, and mounted to protect slides during transportation.

As thick films are easily damaged, five replicates were prepared for each drug concentration and 3 replicates then counted for each experimental condition. Counting was in a blinded fashion to avoid bias. Thick films were counted according to the WHO Mark III protocol. At least 200 asexual parasites were counted. Fields of view were consecutive, starting at the left edge of each blood film and moving stepwise in a uniform direction (to ensure no overlap). Asexual parasites were grouped into either trophozoites or schizonts (defined as asexual parasites displaying 1–2 nuclei and 3 or more nuclei, respectively).

### Growth assay measuring *Plasmodium* lactate dehydrogenase (*p*LDH)

Rhesus monkey red blood cells for *in vitro P. knowlesi* culture were obtained under protocols approved by the independent institutional ethical committee (DEC) according to Dutch and European laws.

The efficacy of the anti-malarial compounds was also assessed *in vitro* against the laboratory maintained *P. knowlesi* H strain [10,11], by measuring the production of *p*LDH [12]. *p*LDH catalyzes the conversion of 3-acetylpyridine adenine dinucleotide (APAD) and lactate to APADH and pyruvate. The enzyme diaphorase subsequently converts nitro blue tetrazolium (NBT) to nitro blue formazan (NBF), using APADH as a reducing agent. NBF can be measured at a wavelength of 655 nm. Serial dilutions of the anti-malarial compounds (100 μl in culture medium) and including a no drug control were dispensed into 96-well plates, to which tightly synchronized (by alanine lysis) early ring-stage infected erythrocytes (100 μl at 2% haematocrit and 2% parasitaemia in culture medium) were added. The plates were then placed in 3% O_2_, 7% CO_2_, 90% N_2_ at 37°C for 22 h to mature (just prior to parasite release). Growth was halted and drug removed, by washing twice in ice-cold PBS. Erythrocytes were lysed by freezing at −20°C and thawing. Aliquots of 0.5 mg/ml of NBT, 1 U/ml diaphorase and 50 μg/ml APAD in LDH buffer (100 mM Tris–HCl, pH 8.0, 50 mM Na L-lactate, 2.5% *v/v* Triton X-100) were added to the thawed cell pellets and incubated for 30 min in the dark at room temperature with shaking. The optical density (OD) at 655 nm of each well was measured in a BioRad 680 microplate reader. OD_655_ values were used to calculate growth by comparing the OD_655_ values at each drug concentration with that of the no drug control. To test the efficacy of artemisinin, artemether, artesunate and DHA, preparations of final concentrations of 0.1 nM to 25 nM were used, and for chloroquine and mefloquine, final concentrations of 1.25 nM to 1 μM were used.

### IC_50_ values and sequence alignments

Dose–response data were modeled using a four-parameter fit and a variable slope, using Prism (Version 4.0a). Goodness of fit was assessed by R^2^, and either noted in the text or highlighted if < 0.8 in tables and figures. For the schizont maturation assay, the top parameter was constrained to 100% for assays using artemisinins but not for mefloquine or chloroquine. For the *p*LDH assay, the top and bottom parameters were constrained to 100 and 0, respectively, for all drugs and, for the ^3^H-hypoxanthine incorporation assay, the top parameter was constrained to 100. Data are summarized as a mean and 95% CI for replicates of single parasite assays and a mean ± SEM for results from multiple independent parasite assays.

The *P. knowlesi* and *P. vivax* orthologues of *P. falciparum* CRT, MDR1, and ATP6 (proteins associated with modulating sensitivities to chloroquine, mefloquine and artemisinins [13]), were aligned with *P. falciparum* 3D7 strain, as a drug sensitive control. H strain and Sal-1 strain sequences were used for *P. knowlesi* and *P. vivax*, respectively. Wild-type and polymorphism sequence data were taken from PlasmoDB [14]. Alignments were performed in MacVector (version 11.0.2).

## Results

### Patient recruitment

Patient isolate data from those obtained in Sarikei and Sibu hospitals, between March and September 2010, are shown in Table 1. A total of nine patients were recruited into this study, six with *P. knowlesi*, two with *P. vivax* and one with *P. falciparum* monoinfections. Infections were initially diagnosed by microscopy and confirmed later by nested PCR [2,6].

### Parasite development

*In vitro* development data for each isolate used during growth assays are shown in Table 1. *P. knowlesi* field isolates were seeded into growth assays with a starting parasitaemia ranging from 0.4 to 7%. The percentage of schizonts present at the start of the assays ranged from 6 to 33%. Good *in vitro* development was demonstrated for all *P. knowlesi* field isolates, with 53 to 94% of asexual parasites reaching the schizont stage, over a development period of 12 to 17 h. Development was similar for *P. falciparum* and *P. vivax* field isolates (Table 1).

### Growth assays: schizont maturation

**Figure 1:**
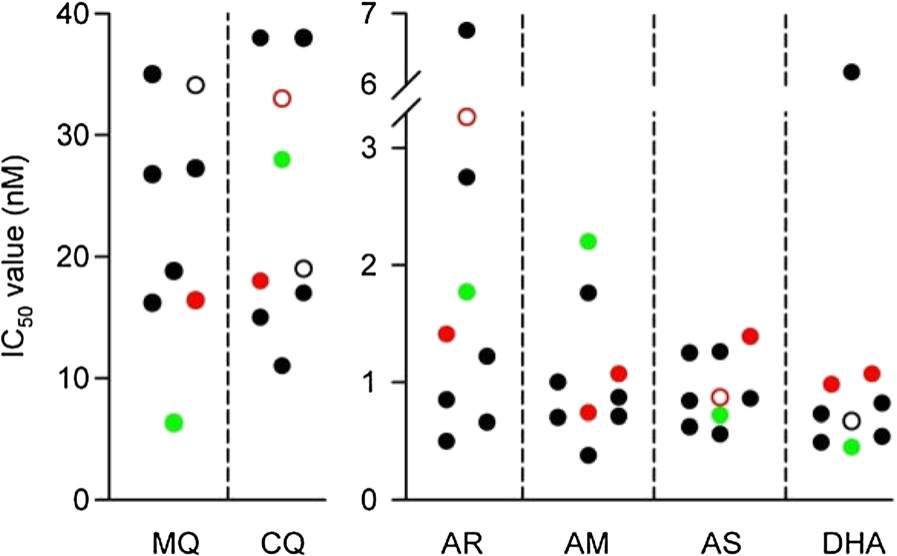
**IC**_**50 **_**values for anti-malarial drugs against *Plasmodium *isolates, using the schizont maturation assay.** Data points represent mean IC_50_ values (nM) derived from single experiments performed in triplicate. *Plasmodium knowlesi* (black symbols), *P. vivax* (red symbols) and *P. falciparum* (green symbols). Filled symbols indicate R^2^ values ≥ 0.8, with unfilled symbols indicting R^2^ values < 0.8. Mefloquine, MQ; Chloroquine, CQ; Artemisinin, AR; Artemether, AM; Artesunate, AS; Dihydroartemisinin, DHA. Note that there is only mefloquine data for one of the two *P. vivax* isolates.

Dose–response curves were used to derive IC_50_ values, which are presented in Figure 1, together with mean values in Table 2. All the isolates from humans were sensitive to artemisinin and its derivatives, with IC_50_ values in the low nM range (from 0.38 to 6.8 nM). The IC_50_ values for chloroquine on *P. knowlesi* from humans were relatively high, with values ranging from 11 to 38 nM but were similar to those derived for the single *P. falciparum* isolate (28 (20 to 38) nM; R^2^ = 0.93) and the two *P. vivax* isolates (33 (10 to 110) and 18 (11 to 27) nM; R^2^ = 0.45 and 0.95, respectively) that were studied.

**Table 2:** ***P. knowlesi *** **drug sensitivity data**

**Drug**	**IC**_**50**_**values (nM) for *P. knowlesi *** **isolates (maturation assay)**^**a**^	**IC**_**50**_**values (nM) for *P. knowlesi *** **H strain ( *p *** **LDH assay)**^**b**^
Mefloquine	26 (± 3.1)	25 (7.4 to 81)^c^
Chloroquine	23 (± 4.8)	3.2 (2.2 to 4.7)
Artemisinin	2.1 (± 0.99)	0.80 (0.35 to 1.9)^c^
Artemether	0.90 (± 0.19)	0.84 (0.34 to 2.1)^c^
Artesunate	0.90 (± 0.12)	2.0 (0.93 to 4.2)^c^
DHA	1.6 (± 0.92)	0.79 (0.62 to 1.0)

^a^Mean (± SEM), from 6 independent experiments (each of which was performed in triplicate).

^b^Mean (95% CIs), from a single experiment repeated in quintuplicate.

^c^R^2^ < 0.8.

IC_50_ values are presented for six anti-malarials on *P. knowlesi* field isolates and laboratory H strain.

*Plasmodium knowlesi* isolates were least sensitive to mefloquine, with a mean IC_50_ value of 26 nM (Table 2). The IC_50_ value for mefloquine on the single *P. falciparum* field isolate (6.3 (2.5 to 16) nM; R^2^ = 0.97) was four-fold less than that of the average *P. knowlesi* IC_50_ value and 2.5-fold less than the lowest *P. knowlesi* IC_50_ value (16 (12 to 22) nM; R^2^ = 0.96).

### Growth assays: *p*LDH

To validate the results of the drug assays on *P. knowlesi* from patients, anti-malarials were assessed against the well characterized *P. knowlesi* H strain grown *in vitro* in rhesus erythrocytes. To determine whether data generated by the *p*LDH method (as used to study the H strain) would be comparable with the adapted schizont maturation method (as used in the field study) a parallel study was performed, using artesunate. The IC_50_ value for artesunate against the H strain was 2.0 (0.93 to 4.2) nM (R^2^ = 0.39), using the *p*LDH assay (from a single experiment repeated in quintuplicate), and 1.2 (0.88 to 1.6) nM (R^2^ = 0.95), using the schizont maturation assay (from a single experiment repeated in triplicate), demonstrating that the two assay methods are comparable, at least in the case of artemisinins. Further IC_50_ values derived from *p*LDH assays are presented in Table 2. All IC_50_ values for field isolates and the *P. knowlesi* H strain for artemisinins are highly comparable.

For mefloquine, the IC_50_ value against the laboratory H strain (25 (7.4 to 81) nM; R^2^ = 0.40) was nearly identical to the mean IC_50_ value of human isolates (26 nM). The IC_50_ values suggest that *P. knowlesi* may be intrinsically insensitive to mefloquine. To ensure that the mefloquine stock used in field studies had not degraded during the study, an aliquot was tested after shipping it back to St. George’s, University of London after the study. Using hypoxanthine incorporation as a measure of growth [15] in a single assay (performed in quadruplicate) with *P. falciparum* (3D7) parasites cultured *in vitro* in human erythrocytes, the IC_50_ value was 9.7 (5.5 to 17) nM (R^2^ = 0.89), confirming that potency of mefloquine was maintained.

Interestingly, the H strain was over 7-fold more sensitive to chloroquine than the average value for field isolates, having an IC_50_ value of 3.2 (2.2 to 4.7) nM (R^2^ = 0.86). This value was also well below the lowest calculated IC_50_ value (11 (5.3 to 24) nM; R^2^ = 0.94) for a field isolate.

### Sequence alignments

**Figure 2:**
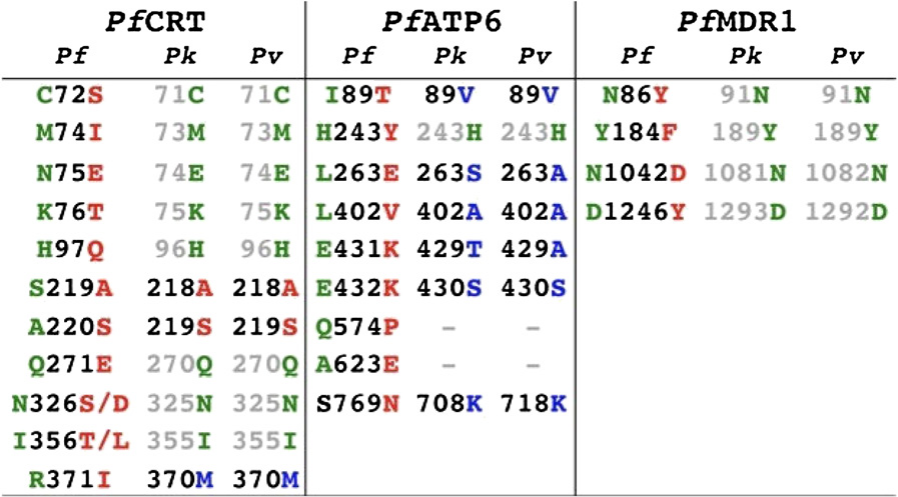
**Alignment of CRT, ATP6, and MDR1 homologues of *P. knowlesi *H strain and *P. vivax *Sal-1 strain, against *P. falciparum *3D7 and strains with known point mutations associated with drug resistance.** Amino acids are colour coded, with green representing the drug sensitive *P. falciparum* 3D7, and red representing amino acid changes found in *P. falciparum* isolates that have a change in drug sensitivity.

Point mutations associated with change in the sensitivity of *P. falciparum* to chloroquine, mefloquine and the artemisinins [13] were analysed, and similarities and differences in sequences encoded by *P. knowlesi* and *P. vivax* homologues are reported in Figure 2. Known loci at which mutations can reduce drug sensitivity were highly conserved between *P. knowlesi* H, *P. vivax* Sal-1 MDR1 homologues and MDR1 in drug sensitive *P. falciparum* 3D7. PfATP6 orthologues demonstrated polymorphism in amino acids in about two thirds of the residues previously examined for effects on drug sensitivity, including L263 [16-18].

The CRT orthologues were fairly well conserved with the exceptions of S219A, A220S, and R371M in the chloroquine sensitive strain. These differences do not confer chloroquine resistance to the *P. knowlesi* H strain. Agreement in sequences in these polymorphic regions was higher between *P. knowlesi* H and *P. vivax* Sal-1 than between either strain and *P. falciparum* 3D7 consistent with a closer phylogenetic relationship between the former species [19].

## Discussion

*In vitro* culture of *P. knowlesi* has only recently been achieved in human erythrocytes [20,21]. However, drug sensitivity assays of natural human infections can only be assessed in short term cultures. These allowed the successful application of drug sensitivity micro-assays such as the WHO Mark III micro-assay test after adaptation to the quotidian life cycle of the parasite and frequent (every 2 to 2.5 h) monitoring of development to allow assessment of when most parasites were mature schizonts in control samples.

The excellent *in vitro* efficacy of the artemisinins against both human *P. knowlesi* (mean IC_50_ values < 2.2 nM) and the laboratory (H) strain is consistent with recent observations made on patients treated with artesunate, where no mortality was observed after treatment [22]. These results also agree with data from animal models, which demonstrate the successful clearance of *P. knowlesi* parasites from infected rhesus monkeys, by artemisinin in combination therapy with naphthoquine [23]. In addition, William *et al.*[24] in a retrospective analysis of clinical cases of knowlesi malaria in Sabah, reported the successful treatment of patients by artemether-lumefantrine combination therapy. This study also noted that where knowlesi malaria developed signs of severity, intravenous artesunate was effective [24].

Intriguingly, chloroquine IC_50_ values from this field study, including those derived against *P. knowlesi* (with values ranging from 11 to 38 nM), were higher than that of the laboratory *P. knowlesi* H strain (3.2 nM), although the reason for this is unclear. In general, the values are comparable with chloroquine-sensitive laboratory strains of *P. falciparum*, having IC_50_ values ranging from 8 to 15 nM [25-27]. These values all fall below the 100 nM threshold used to define chloroquine resistance [27,28] and there is no evidence for clinical chloroquine resistance reported in *P. knowlesi*. Chloroquine is effective both as a monotherapy and when used in combination with primaquine [1,24,29]. Consistent with these findings, Tyagi *et al.*[30] have recently reported that CRT (and DHFR) sequences from *P. knowlesi* clinical isolates collected in the Andaman and Nicobar Islands, India, were all found to be wild-type (with close homology to the CRT sequence of chloroquine sensitive *P. falciparum* parasites – see Figure 2 and below). Nevertheless, continued monitoring of chloroquine sensitivity in *P. knowlesi* might be prudent bearing in mind the history of chloroquine resistance development in other malarial species.

Evidence suggests that transmission of *P. knowlesi* to humans in Sarawak remains zoonotic and, thus, ostensibly free from mefloquine drug selection pressure. However, results with mefloquine consistently showed a low sensitivity in *P. knowlesi* field isolates, when compared with that of *P. falciparum*. The mean IC_50_ value for mefloquine calculated for the 6 *P. knowlesi* isolates is 26 nM, which is just above the value used to define mefloquine resistance in *P. falciparum* (>24 nM) in some reports [31,32] but well below that reported by others (>119 nM) [33]. Importantly, the reduced *P. knowlesi* response in human isolates was also observed in the *P. knowlesi* H experimental line. Given the efficacy of the mefloquine used was confirmed after the end of the study, these results indicate an innate tolerance of *P. knowlesi* to mefloquine. These findings also suggest the strong possibility of treatment failure if mefloquine is used as mono or combination therapy for *P. knowlesi* and is supported by reports of mefloquine treatment failure in rhesus monkeys infected with *P. knowlesi*[34], as well as recent cases of mefloquine treatment failure in humans with knowlesi malaria [35]. On this basis, mefloquine should be used with caution for the treatment of knowlesi malaria, or indeed for prophylaxis against malaria in areas where acquiring knowlesi is a risk until larger studies have been undertaken.

There is reasonable conservation of MDR1 sequences between *P. vivax* Sal-1 and *P. knowlesi* H and the *P. falciparum* 3D7 reference strain. Previous studies on *P. falciparum* found that increased *mdr1* copy number conferred a mefloquine resistant phenotype [36] and risk of treatment failure, although *P. knowlesi mdr1* copy number in the isolates reported in the current study have not been determined. Alignments of the *P. vivax* Sal-1 and *P. knowlesi* H CRT and ATP6 orthologues with the *P. falciparum* 3D7 sequence revealed several polymorphic differences between the *P. vivax* and *P. knowlesi* alignments and that of the *P. falciparum*. These substitutions do not alter sensitivity of *P. knowlesi* to artemisinins and give insights into the possible contributions of these residues to artemisinin sensitivity in *P. falciparum*.

Here, it has been established that it is possible to culture *in vitro P. knowlesi* in human erythrocytes in the short term, when taken *ex vivo*. In addition, the successful adaption of the schizont development assay to determine anti-malarial drug sensitivities of *P. knowlesi* field isolates has been shown. Using this adapted method, this study has demonstrated that chloroquine and artemisinin based drugs are effective against *P. knowlesi* parasites. Conversely this study has shown poor sensitivity of *P. knowlesi* field isolates and laboratory H strain to mefloquine, suggesting innate reduced sensitivity of the parasite to this important anti-malarial drug.

## Competing interests

The authors declare that they have no competing interests.

## Authors’ contributions

The study was conceived by SK and designed by HMS, CHMK, BS, JCS and SK. The assays were performed by FAF with support from HMS, AS, MAA, LCW, JCS and EMP. The manuscript was prepared by FAF, HMS, and SK. All authors had the opportunity to read and approve the manuscript.
